# Modifying the Tumour Microenvironment: Challenges and Future Perspectives for Anticancer Plasma Treatments

**DOI:** 10.3390/cancers11121920

**Published:** 2019-12-02

**Authors:** Angela Privat-Maldonado, Charlotta Bengtson, Jamoliddin Razzokov, Evelien Smits, Annemie Bogaerts

**Affiliations:** 1 PLASMANT, Chemistry Department, University of Antwerp, 2610 Antwerp, Belgium; charlotta.bengtson@uantwerpen.be (C.B.); jamoliddin.razzokov@uantwerpen.be (J.R.); annemie.bogaerts@uantwerpen.be (A.B.); 2 Solid Tumor Immunology Group, Center for Oncological Research, University of Antwerp, 2610 Antwerp, Belgium; evelien.smits@uantwerpen.be

**Keywords:** cold atmospheric plasma, cell communication, extracellular matrix (ECM), reactive oxygen and nitrogen species (ROS), tumour microenvironment (TME), extracellular vesicles, communication junctions, three-dimensional in vitro culture models

## Abstract

Tumours are complex systems formed by cellular (malignant, immune, and endothelial cells, fibroblasts) and acellular components (extracellular matrix (ECM) constituents and secreted factors). A close interplay between these factors, collectively called the tumour microenvironment, is required to respond appropriately to external cues and to determine the treatment outcome. Cold plasma (here referred as ‘plasma’) is an emerging anticancer technology that generates a unique cocktail of reactive oxygen and nitrogen species to eliminate cancerous cells via multiple mechanisms of action. While plasma is currently regarded as a local therapy, it can also modulate the mechanisms of cell-to-cell and cell-to-ECM communication, which could facilitate the propagation of its effect in tissue and distant sites. However, it is still largely unknown how the physical interactions occurring between cells and/or the ECM in the tumour microenvironment affect the plasma therapy outcome. In this review, we discuss the effect of plasma on cell-to-cell and cell-to-ECM communication in the context of the tumour microenvironment and suggest new avenues of research to advance our knowledge in the field. Furthermore, we revise the relevant state-of-the-art in three-dimensional in vitro models that could be used to analyse cell-to-cell and cell-to-ECM communication and further strengthen our understanding of the effect of plasma in solid tumours.

## 1. Introduction

Organs are the structural and functional units of the body composed by cells responsible for their particular function (e.g., enzyme secretion) and the stroma (supportive framework formed by stromal cells and extracellular matrix (ECM)). In cancer, solid tumours resemble organs with abnormal function and structure that unlike normal organs, can have detrimental effects on the survival of the individual. In fact, the multiple cellular (endothelial cells, fibroblasts, inflammatory cells, immune cells) and acellular components (ECM elements and secreted factors), collectively termed the ‘tumour microenvironment’ (TME), play an active role in the survival, growth, invasion, and metastasis of cancer cells. Cancer research has long focused on the development of therapies against tumour cells; however, it is now acknowledged that the TME plays a key role in modulating the progression of tumour growth and resistance to chemotherapeutic drugs [1]. Changes in the TME are transmitted to cancer cells due to the dynamic and interdependent interaction between cells and TME components. This communication involves direct physical cell-to-cell interactions (via gap, tight and anchoring junctions, among others), indirect communication via secreted signals (cytokines, growth factors), and cell-to-ECM interaction via binding of transmembrane adhesion proteins (cadherins, integrins) with ECM components. Novel cancer therapies targeting one or more of the TME components could be beneficial to control and eliminate tumours and could overcome the limitations of current treatments. An emerging technology from the field of physics, called ‘plasma’, presents as an innovative anticancer approach, due to its potential to eliminate cancer cells and to activate specific signalling pathways involved in the response to treatment.

Plasma is the fourth state of matter and it can be generated by coupling sufficient quantities of energy to a gas to induce ionization [2]. During ionization, the atoms or molecules lose one or several electrons, resulting in a mixture of free electrons and ions, called “ionized gas”. The free electrons can furthermore cause excitation and dissociation of the atoms or molecules, resulting in the generation of a mixture of neutral, excited, and charged species that exhibit collective behaviour [3]. Cold plasma (hereinafter simply referred to as ‘plasma’) is of particular interest in biomedicine. The high temperature of the electrons determines the ionization and chemical processes, but the low temperature of heavy particles determine the macroscopic temperature of plasma [4]. Plasma can be generated at atmospheric pressure and body temperature, below the tissue thermal damage threshold (43°C) [3,5,6,7]. Biomedical plasmas can (mostly) be classified into two groups: dielectric barrier discharge (DBD) devices that generate plasma in ambient air, and plasma jets that first ionize a carrier gas that later interacts with molecules present in ambient air. In DBDs, plasma is generated between a powered electrode (covered by an insulating dielectric material) and the target (tissue or sample) that operates as the second electrode, placed in close proximity. The dielectric material accumulates the charge that helps sustaining the generation of plasma, and reduces the current passed into the tissue to generate a thermally and electrically safe plasma [8]. In the plasma jet configuration, the system is fed by a constant gas flow (argon, helium, nitrogen) that is ionized around the powered electrode inside the device. As the ionized gas is transported outside in propagating ionization waves, it forms a stream of active particles discharging as a jet that can extend up to centimetres away from the device [9]. Plasma reacts with oxygen and nitrogen molecules present in ambient air to form an assortment of reactive oxygen species, such as hydrogen peroxide (H_2_O_2_), hydroxyl radical (•OH), superoxide (O_2_•^‒^), ozone (O_3_), singlet delta oxygen (^1^O_2_), and atomic oxygen (O), as well as reactive nitrogen species (RNS), such as peroxynitrite (ONOO^‒^), nitrogen dioxide radical (•NO_2_) and nitric oxide (•NO) [10,11,12]. This group of reactive species is collectively referred from hereon as ROS, due to the presence of oxygen in all the biologically relevant RNS.

ROS are largely considered as the main agents responsible for the biological effects exerted by plasma in cells and tissues [7,13,14], while other physical components, such as electromagnetic fields and UV photons, do not significantly contribute to the overall effect on cells individually at the levels generated by plasma [15,16,17]. Plasma technology offers the possibility to induce different biological responses in cells and tissues (e.g., wound healing, coagulation, elimination of cancerous cells) which is dependent on the nature, location and levels of ROS produced [18]. These effects are believed to be due to the combination of the direct interaction of plasma-derived ROS with cells (some ROS are able to penetrate to up to several mm into the tissue, reviewed in [19]) and the consequent generation of signals that modify the microenvironment at further distances and that activate an immune response. The effects in cells and tissues can be obtained by delivering plasma directly (plasma rich in short- and long-lived ROS) or indirectly (plasma-treated solutions rich in long-lived ROS used for treatment), which adds to the versatility of plasmas [19]. This is particularly important, considering that indirect treatments could favour the delivery of plasma-derived ROS to hard-to-reach regions of the body where direct plasma treatments cannot reach.

Cancer cells constitutively present higher levels of ROS than normal cells due to alterations on their metabolism, genomic instability, mitochondrial disfunction and TME modifications [20,21]. This makes cancer cells more vulnerable to high ROS levels, increasing their sensitivity and apoptosis from increased ROS [22]. Current anticancer therapies such as radiotherapy induce intracellular ROS formation through radiolysis of water and secondary reactions to damage the DNA and lead to cell death [23]. In the same way, a large number of chemotherapeutic drugs with cytostatic and cytotoxic activity eliminate cancer cells by generating high levels of intracellular ROS [24]. However, the sensitivity to treatment is affected by the local levels of O_2_ required for ROS formation [25] and the intracellular pathways affected. It has been suggested that therapies providing external ROS, such as plasma, could raise the threshold beyond which cell death can be induced in cancer cells without harming normal cells [26,27]. In addition, the localized application of plasma could limit the exposure of healthy tissue to ROS, an advantage over radiotherapy and chemotherapy.

Multiple studies have demonstrated the cytotoxic effect of plasma on cancer cells using in-house built, standard, and commercial plasma devices [9,28,29]. The main mechanisms of action involve the induction of apoptosis, cell cycle arrest and inhibition of cancer cells dissemination [30,31,32,33,34,35]. To date, most of the research on plasma treatments for cancer has been focused on malignant cells, even though ever more often the TME has been shown to contribute significantly to tumour progression and to the response to chemotherapy [1,36]. Considering that tumours are complex organs formed by more than cancer cells, it would be expected that plasma-derived ROS would react with all the cellular and acellular components of the TME. This observation raises some questions: Does the oxidation of ECM components and binding proteins affect the proliferation, survival, and migratory abilities of cancer cells? How does plasma affect the endothelial cells, fibroblasts and resident immune cells present in the tumour? Do plasma-derived ROS participate as messengers of cell communication? Which mechanisms of communication are involved in the propagation of the plasma treatment effect at distant places from the treatment site? There is a limited body of literature about the effect of plasma on the TME and its role in the communication of cells with their surroundings. Understanding the major events occurring in the tumour upon plasma treatment and how does plasma modulate the communication between cancer cells and the TME is of outmost relevance to develop efficient plasma therapies for cancer.

This review aims to discuss the current state of the field on plasma treatments in the context of the TME, considering the cell-to-cell and cell-to-ECM interactions affected by plasma in tumours. Additionally, we will discuss relevant three-dimensional (3D) in vitro strategies to explore the effect of plasma on solid tumours, especially considering the role of the TME. As our understanding of the mechanisms of action of plasma in cancer cells continues to expand, it is necessary to consider the complexity of solid tumours to develop more efficient plasma therapies.

## 2. Plasma-Derived ROS, Cell Death, and the ECM

A significant number of studies have been done to identify the type and spatio-temporal distribution of ROS produced by biomedical plasmas and the reader is referred to [9,37] for details. Although the ROS composition between plasma devices may vary, it has been shown that plasma-derived ROS can oxidize lipids in the cell membrane, reduce the membrane fluidity and favour pore formation [38,39,40], a topic thoroughly reviewed in [19]. The permeabilization of the cell membrane facilitates the access of ROS into the intracellular compartment, as well as the release of cell contents to the ECM, as observed in necrotic cells [41]. In the same way, plasma can induce oxidative stress in membranes of intracellular organelles [42]. The transport of plasma-produced H_2_O_2_ is also favoured by the increased number of aquaporins present in the plasma membrane of cancer cells [43]. Furthermore, plasma affects the different proteins forming the ECM, in the plasma membrane or inside the cells. Changes to the function or structure of ECM proteins or at the cell surface can activate signalling pathways to alter gene expression, cell growth and maintenance [44]. Within the cell, plasma-derived ROS can oxidize proteins involved in metabolic pathways, proteasome activity and mitochondrial respiration [42]. In addition, plasma can cause double-strand DNA breaks [42,45,46] that if irreversible, can lead to cell death [47].

There is a growing body of evidence suggesting that the anticancer effect of plasma is predominantly caused by apoptosis-induction mediated by ROS [48,49,50,51,52,53,54]. These ROS will primarily act in the ECM, even though there are studies verifying the importance of a rise of intracellular ROS levels in successful plasma treatment of cancer cells [31,48,55,56,57]. The origin of the ROS triggering apoptosis in cancer cells after plasma treatment has been under debate: Although it would be logical to assume that the ROS were generated by plasma and added exogenously, it is true that some of these ROS have a very short life time and diffusion length due to their highly reactive nature and will not be able to reach the ECM, particularly not in the bulk of a cancer tumour. A paradigm first proposed in [58] and recently experimentally verified [59,60], states that the ROS acting in the ECM of cancer cells after plasma treatment instead are generated by the cells themselves and are part of a system of signalling pathways used by cancer cells to promote proliferation. In this scenario, the effect that plasma exerts on cancer tumour is constituted by the transmission and/or creation of one species, ^1^O_2_, to the ECM. ^1^O_2_ interferes with the system of signalling pathways in such a way that the proliferative signal turns into an apoptosis-inducing signal. This paradigm also accounts for the observed selectivity of plasma treatment [56,57,61,62,63,64,65,66,67,68,69,70], since the signalling pathways (which are introduced in detail below) manipulated by plasma do not exist in normal cells. However, it is worth considering the effect of cell type, cancer type, and culturing medium on the response observed before selectivity can be claimed, as recently demonstrated [71].

One of the main differences between cancer cells and normal cells that enables a selective effect of plasma on cancer over normal cells is: a) the generation of O_2_•^‒^ into the ECM and b) the presence of catalase associated to the external surface of the cell membrane. The generation of O_2_•^‒^ is found already in transformed cells, i.e., yet not fully developed cancer cells, and is required for their proliferation [72,73,74,75,76,77,78,79,80,81,82,83]. However, at this stage, O_2_•^‒^ is also detrimental for the cells since O_2_•^‒^ furthermore functions as the species from which two apoptosis-inducing signalling pathways originate. These signalling pathways are the HOCl pathway [84,85,86,87] and the ONOO^‒^ pathway [84,85,87,88], respectively, named after the species in each pathway, from which the highly reactive •OH is formed. When •OH is formed in the vicinity of the cell membrane, it causes lipid peroxidation and subsequent cell death by apoptosis. The extremely short lifetime and diffusion length of •OH prevent harm on adjacent cells. Other factors that could contribute to the selective effect of plasma on cancer cells are the high steady-state of intracellular ROS produced [89], the increased number of aquaporins in the plasma membrane that can transport H_2_O_2_ into the cells [43] and the reduced levels of cholesterol in the cell membrane of cancer cells that favours the penetration of ROS [38].

**Figure 1:**
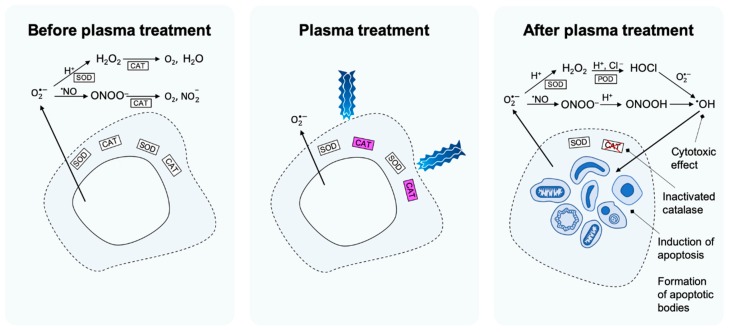
Proposed set of events underlying the mechanism of apoptosis induction in selective plasma cancer treatment. Before plasma treatment, catalase prevents formation of •OH in the extracellular matrix (ECM) from the HOCl and the ONOO^‒^ pathway. In the HOCl pathway, this occurs through decomposition of H_2_O_2_ and in the ONOO^‒^ pathway it precedes through decomposition of ONOO^‒^. During plasma treatment, catalase is inactivated by ^1^O_2_ that is either contained in the plasma or generated in the ECM from components transferred from the plasma. After plasma treatment, the cancer cell’s protection against the HOCl and the ONOO^‒^ pathway is lost and •OH is formed in the ECM. Through lipid peroxidation in the cell membrane, •OH is causing apoptosis induction.

For a transformed cell to reach the state of a cancer cell, it needs to protect itself from the apoptosis-inducing signalling pathways, which is achieved by relocating intracellular catalase into the outer cell membrane. Indeed, studies show that membrane-associated catalase is crucial for cancer cell progression [84,85,90,91,92,93,94,95]. Catalase decomposes H_2_O_2_ as well as ONOO^‒^ and thus, removes the substrates for production of •OH in both the HOCl pathway and ONOO^‒^ pathway (Figure 1). In addition, when H_2_O_2_ is not decomposed, it may, as already mentioned, also enter the intracellular compartment through aquaporins in the cell membrane, where it causes depletion of glutathione [59,96]. The depletion of intracellular glutathione renders the cells more sensitive for •OH attacking the membrane.

Depending on the plasma treatment conditions, ^1^O_2_ could either be generated directly and transferred from the gaseous phase to the liquid phase of the ECM or generated indirectly from H_2_O_2_ and NO_2_^‒^ (which are transferred from the gaseous phase to the ECM). The latter scenario, where so-called ”primary” ^1^O_2_ is generated in the ECM, has been elucidated to be the most likely [59,60]. Indeed, the formation of such primary ^1^O_2_ from a solution of H_2_O_2_ and NO_2_^‒^ has been experimentally verified [96,97]. Since ^1^O_2_ has the capacity to inactivate catalase through reaction with histidine in the active centre of the enzyme [98], application of plasma to a cancer cell will thus lead to a loss of the protection against the HOCl and ONOO^‒^ pathway, which causes the cancer cells to undergo apoptosis. However, the generation of primary ^1^O_2_ has been found to be below the detection level [97]. Nevertheless, the concentration has been found to be sufficient to inactivate a few membrane-associated catalase molecules. In the vicinity of the sites where catalase has been inactivated, the non-decomposed H_2_O_2_ and ONOO^‒^ will not only enter the apoptosis-inducing signalling pathways (the HOCl and ONOO^‒^ pathway) and cause intracellular glutathione depletion (in the case of H_2_O_2_), but they are also the substrates in a set of reactions where ^1^O_2_ is generated [99,100,101,102]. This so called “secondary” ^1^O_2_ may subsequently inactivate catalase in the cell membrane of the same cell, or the catalase in the cell-membrane of adjacent cancer cells. The generation of secondary ^1^O_2_ occurs in an exponential manner and creates an amplified apoptosis signal reaching the bulk of the tumour.

The whole set of events—from the generation of primary ^1^O_2_, catalase-inactivation, and subsequently, tumour-generated secondary ^1^O_2_ (followed by inactivation of more catalase), to apoptosis-induction—has been experimentally investigated and verified both in the case of tumour cells in a solution of H_2_O_2_ and NO_2_^‒^ [96,97] and for plasma-treated tumour cells [59,60]. The selectivity of the treatment, given that the concentration of H_2_O_2_ is below a certain limit, has been confirmed by control experiments with non-malignant cells [59].

A question that arises when considering the paradigm postulated in [58], is whether or not there is an explicit relationship between the parameters of the plasma treatment (e.g., ROS composition and treatment duration) and the concentration of •OH formed to induce apoptosis. The relationship between the extent of catalase inactivation and the resulting generation of •OH from the HOCl and ONOO^‒^ pathways was investigated theoretically by mathematical modelling of the reaction kinetics in [103]. In this context, cancer cells would need very high concentrations of catalase (in the mM order) to protect themselves from the ONOO^‒^ pathway, which was the only pathway found to generate •OH in a significant amount. In order for a substantial generation of •OH, most of this catalase (about 99%) has to be inactivated.

## 3. The Tumour Microenvironment (TME)

**Figure 2:**
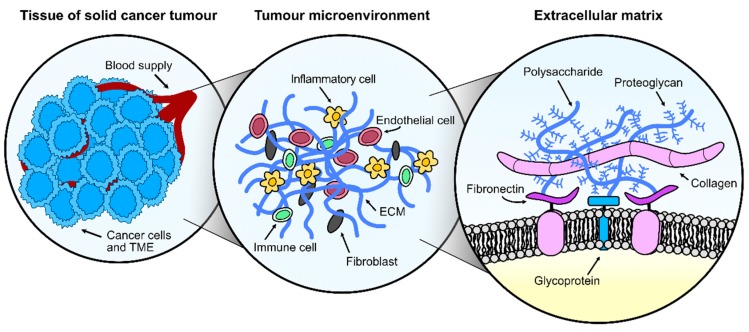
Understanding the complexity of solid tumours for anticancer plasma treatment. A solid cancer tumour consists of cancer cells as well as several other components in constant interaction, collectively referred to as the tumour microenvironment (TME). The TME is formed by endothelial cells, fibroblasts, inflammatory cells, immune cells in addition to extracellular matrix (ECM) components, most prominently collagen, fibronectin, polysaccharide chains, glycoproteins, and proteoglycans. All TME components are susceptible to plasma-derived reactive oxygen and nitrogen species (ROS) and their response to plasma could affect the treatment outcome.

The TME consists of malignant and non-transformed cells, tumour vasculature and ECM, all in constant interaction. Non-malignant cells in the TME dynamically participate in all stages of carcinogenesis where they often have a tumour-promoting function [104]. The close communication between cells and ECM via a dynamic network of soluble factors, such as cytokines, chemokines, growth and angiogenic factors and enzymes, orchestrate the uncontrolled cell growth, resistance to cell death, hypoxia, and dysplasia in tumours. In addition, this interaction is required for the formation of new blood and lymph vessels, stroma remodelling, recruitment of immune cells and cancer-associated fibroblasts, and metastatic processes [1]. The TME presents multiple components that could interact with plasma-derived ROS and alter the response evoked in the treated tumour cells (Figure 2), therefore it is critical to identify how plasma affects the TME and how we could modulate these responses to obtain better therapeutic outcomes.

### 3.1. Cellular Components of the Tumour Microenvironment

The TME contains a heterogeneous mass of malignant cells, immune cells, endothelial cells, and fibroblasts. During wound healing, fibroblasts acquire a myofibroblast state that promotes ECM remodelling, epithelial proliferation and angiogenesis. In fact, plasma treatments for wound healing have shown to accelerate the healing process by promoting fibroblast activation, migration and proliferation [105], secretion of angiogenesis-related molecules (angiogenin, endostatin, MCP-1, EG-VEGF, artemin and FGF-2) [106], activation of PPAR-γ anti-inflammatory pathway [107] and NF-κβ pathway in fibroblasts [108] without inducing their apoptosis [109]. In cancer; however, the actions exerted by activated fibroblasts (termed cancer-associated fibroblasts, CAFs) promote tumour development by initiating ECM remodelling (by the secretion of matrix metalloproteinases, MMPs) and secreting cytokines and growth factors [110]. CAFs are considered critical players in the malignant progression with a complex bidirectional communication mechanism between CAFs and cancer cells mediated by cytokines and RNA transference via exosomes to favour metastasis, vascular permeability, and resistance to chemotherapy and radiotherapy [111,112,113]. It has been proposed that plasma has dual effects on fibroblasts, as short treatments enhanced the cell viability and collagen production, whereas longer treatments inhibit them [114]. Exposure to longer plasma treatments of higher plasma-derived ROS concentrations can induce senescence [115] and necrotic cell death in fibroblasts [116]. As CAFs are recognized as important targets for cancer treatment, we should consider whether the elimination or modulation of CAFs activity by plasma is possible to assist tumour control.

Tumours recruit their own vasculature for a constant supply of oxygen and nutrients, removal of waste products and escape routes to enable tumour metastasis [117]. Vascular endothelial growth factors (VEGFs) are secreted to promote the formation of new blood vessels via sprouting, intussusception, vasculogenic mimicry or mosaic vessel formation [118]. The excessive proliferation of endothelial cells leaves the vasculature poorly covered by perivascular cells needed for vasoconstriction and vasodilation [117] and the intercellular spaces formed permit the free pass of macromolecules and tumour cells (metastasis) [104]. In addition, the leakage of blood plasma into the interstitial tissue reduces the blood flow velocity, which causes occlusion of the blood vessels, acute hypoxia, and continuous release of VEGF [118,119]. To alleviate the pressure inside the tumour and drain the excessive fluid from the interstitial tissue, malignant cells recruit their own lymphatic system [120]. In wound healing, plasma has shown to promote paracrine and autocrine signalling via angiogenic factors, such as angiopoietin-2, angiostatin, endostatin, amphiregulin and FGF-2 produced by keratinocytes, fibroblasts, and endothelial cells that favoured tube formation [106]. In this study, HUVEC endothelial cells were more sensitive to plasma than keratinocytes and fibroblasts, as they presented higher levels of double-strand DNA damage. Indeed, higher plasma-derived ROS levels can induce cell cycle arrest, reduced cell motility and DNA damage [121], which supports the idea that the elimination of both endothelial and cancer cells with plasma could aid to control the tumour progression.

Cancer cells promote an immunosuppressive TME to support their growth and evade clearance by the immune system. The main two populations in the tumour immune microenvironment (TIME) are the tumour-associated macrophages and T cells. The infiltration, priming and activation of cytotoxic CD8^+^ T (CTL) and natural killer (NK) cells into the tumour core is facilitated by the secretion of proinflammatory cytokines secreted by T helper-1 (Th1) cells [122]. Without the presence of cytotoxic lymphocytes, tumours remain immunological ignorant and malignant cells cannot be identified by the adaptive immunity [123]. The uncontrolled tumour growth and TME remodelling prevents the immune system to control the tumour progression and favours the recruitment of CD4^+^ T regulatory (Tregs) cells, which supress the priming, activation and cytotoxic activity of effector immune cells, such as Th1 cells, CTL, macrophages, NK cells, and neutrophils, through contact-dependent (PDL-1, LAG-3, CD39/73, CTLA4, or PD1) and contact-independent mechanisms (secretion of IL-10, TGF-β, prostaglandin E2, adenosine, and galectin-1, among others) [122]. Plasma treatments have shown to increase T cell infiltration in murine pancreatic tumours, which could be related to the activation of immunogenic cell death of cancer cells, expressing calreticulin and releasing damage-associated molecular patterns [124]. Additionally, it has been proposed that plasma-derived ROS could upregulate the expression of major histocompatibility complex-I, favouring antigen presentation by cancer cells which could result in an increased number of intratumoural CD8+ T cells [125,126]. In the same way, B cells may play an important role in modulating the tumour response, as they can secrete IL-10 and TGF-β to favour tumour cell proliferation. The antibodies produced by B cells can alter the function of their antigens present on cancer cells, activate the complement cascade, or promote antibody-dependent cell-mediated cytotoxicity [127], as well as stimulate angiogenesis and chronic inflammation that promotes the progression of tumours [128]. To date, only one study has assessed the survival of peripheral blood B cells exposed to plasma in vitro, but how does plasma affect B cells in tumours is yet unknown.

Macrophages are specialized cells able to present antigens to activate T cells and secrete cytokines to activate other cells. Tumour progression and TME modifications favour the differentiation of tumour-associated macrophages (TAM) toward a pro-tumourigenic phenotype (M2-like polarization) which secrete anti-inflammatory cytokines, in contrast to the proinflammatory (M1-like polarization) phenotype that contribute to the elimination of cancer cells [129,130]. TAMs are recruited to the tumour site by CCL2, CCL5, VEGF, and CSF1 and participate in a variety of pro-tumourigenic processes, such as angiogenesis (VEGF), cell proliferation (EGF) and epithelial-mesenchymal transition, tumour metastasis, immunosuppression (IL-10 and TGF-β), ECM remodelling (MMPs), and reduction of anticancer therapies efficacy [122]. Interestingly, it has been shown that plasma-derived ROS influence the differentiation profile of monocytes [131,132] and the inflammatory potential of macrophages [133]. Plasma can induce the polarization towards M1 phenotype of monocyte-derived THP-1 macrophages which secrete the proinflammatory cytokines IL-1α, IL-1β, IL-6, and TNF-α and upregulate the inducible nitric oxide synthase [134,135]. Plasma-activated macrophages display increased mobility [135,136] and tumour infiltration ability [35,124]. In addition, they can reduce the viability, invasive behaviour, and ATP content in cells, inhibit cell growth, and induce cell death by affecting genes involved in DNA damage checkpoints [134].

Dendritic cells (DCs) have an important role in antigen processing and presentation to T cells to evoke an adaptive immune response against malignant cells. However, tumour-associated DCs (TIDCs) are usually associated with immunosuppression due to the high expression of regulatory molecules and receptors, debilitated antigen cross-presentation, and low costimulatory molecule expression [137]. In addition, malignant cells and the TME secrete factors to inhibit or reverse the normal function and maturation process of DCs [138]. Plasma treatment of murine pancreatic tumours did not affect the number of TIDCs [124]. However, in vitro studies have shown that DCs were more prone to phagocytose pancreatic cancer cells exposed to plasma-treated PBS, as these cells expressed and released damage-associated molecular patterns characteristic of immunogenic cell death, favouring maturation of DCs [139]. Neutrophils are also recruited to the TME by CXCR2 ligands secreted by cancer and stromal cells [140] to eliminate cancer cells through the deposit of neutrophil extracellular traps (NETs) and exocytosis of protease-containing granules. It has been proposed that the secretion of NETs (composed by chromatin, MMP-9, elastase, cathepsin G and intracellular proteins) promotes tumour progression and metastasis, as the proteases digest the ECM and facilitate the migration and invasion of cancer cells [141]. Previous studies in the context of wound healing have reported profound NET formation and IL-8 secretion in plasma-treated human neutrophils, which could be detrimental for cancer treatment [142]. Other cells, such as the adipose mesenchymal stromal cells (AMSCs) possess proangiogenic, antiapoptotic, proliferative, and multipotent differentiation characteristics that are often associated with tumour initiation and metastasis [143]. AMSCs have immunosuppressive properties and a positive tropism towards the TME [144] where they can interfere with the maturation of DCs and the proliferation and differentiation of B cells and ECM composition [145]. Plasma has been shown to inhibit adipogenic differentiation [146] and to induce senescence, cell cycle arrest, and M2 macrophage polarization [115]. To date, there is limited information about the effect of plasma on AMSCs in the context of tumours.

It is worth considering that although cancer and immune cells present phenotypic and functional heterogeneity, TIME can be forged by tumour cell-intrinsic factors and in this way, determine the sensitivity to cancer treatments [147]. The existence of cancer stem cells, able to avoid the immune system, metastasize, and resist chemotherapeutical drugs, brings further levels of complexity to the development of successful therapies against cancer. The use of plasma to selectively suppress or eliminate cancer stem cells, to modify the TME that supports their development and proliferation, and to activate the response of the immune system against malignant cells could significantly benefit anticancer therapies. Further studies are needed to determine the effect of plasma treatments in complex solid tumours, considering these variables in the overall response.

### 3.2. Acellular Components of the Tumour Microenvironment

**Figure 3:**
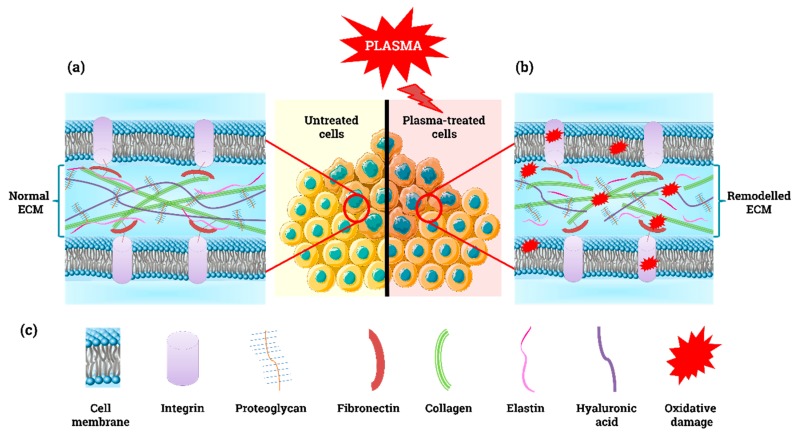
Simple model of the extracellular matrix. (**a**) Normal and (**b**) remodelled ECM under oxidative stress. (**c**) Schematic illustration of ECM components.

Beside cells, the TME comprises a complex 3D architecture formed by collagen, elastin, fibronectin, glycoproteins, and proteoglycans (see Figure 3a,c and Table 1). The ECM elements are responsible for providing support to the tissue, storing growth factors, and controlling tissue stiffness. The TME is in constant architectural modification in response to cell proliferation. The increased deposition of ECM components modifies the biomechanical properties of the ECM, interferes with cell-to-cell adhesion and cell polarity, and enhances growth factor signalling [148]. The disorganized growth of malignant cells, poor tissue oxygenation and increased inflammation in the TME induce desmoplasia (excessive collagen deposition), which restricts the penetration of chemotherapeutic drugs and migration of immune cells towards the cancer cells [149]. The mechanical and chemical changes of the ECM are communicated to cells through integrins located in their cell membrane (Figure 3a,b), which can result in the development of an invasive phenotype [110].

It has been suggested that agents aimed to deconstruct the ECM or to modulate the deposition of ECM components could improve the response of tumours to chemotherapy or other treatments [150,151,152]. In vitro studies have shown that plasma can destroy dry and dissolved collagen I molecules due to the oxidation of amino acids and breakage of hydrogen bonds [153], which loosen the collagen structure (Figure 3b) [154]. In addition, plasma decreased the collagen secretion and cell migration in keloid fibroblasts [155], which similarly to CAFs, overproduce collagen [156]. Using Matrigel (matrix extracted from the Engelbreth-Holm-Swarm mouse sarcoma rich in laminin, collagen IV, entactin, proteoglycans, and growth factors) as a surrogate model in vivo, it was shown that high doses of plasma-derived ROS hindered ECM-cell interactions and decreased bone formation, whereas lower ROS doses promoted chondrocyte differentiation, VEGF production and bone formation [157]. Similarly, mild plasma treatments for skin rejuvenation have shown to significantly enhance the expression of collagen I, fibronectin, and VEGF in fibroblasts to boost angiogenesis and repair of connective tissues [158]. To date, only two clinical studies have reported changes in the ECM of wounds in patients with head and neck cancer after palliative plasma treatment. The desmoplastic reaction induced by plasma suggests an increased deposition of collagen [159,160], a response often associated with impaired drug delivery and penetration of anticancer treatments [161]. However, it is possible to re-educate stromal cells to reduce desmoplasia, as shown for pancreatic stellate cells [162].

Hyaluronan or hyaluronic acid (HA) is a gel-forming CAF-produced ECM proteoglycan that is key in the autocrine and paracrine signalling between CAFs and malignant cells. High HA synthesis is correlated with aggressive behaviour and tumour spreading in different cancer cell types, as HA immobilizes and deactivates monocytes patrolling the tumour, boosts cell proliferation, inhibits apoptosis, and enhances the epithelial-to-mesenchymal transition in cancer cells to activate metastasis [163]. Treatments that target HA and its synthesis could destroy the fibrotic stroma in tumour to allow the delivery of therapeutic agents [164]. Previous studies have shown that ROS can depolymerize and fragment HA aggregates [165] and oxidize other ECM components (Figure 3b), such as fibronectin [166]; however, the effect of plasma on these ECM components is yet to be studied.

## 4. Mechanisms of Cell Communication

### 4.1. Cell-to-Cell Communication

The cytotoxic effect of plasma in cancer cells is not restricted to the direct interaction of plasma-derived ROS and the target cells, as its effect can be seen in cells located beyond the diffusion radius of ROS. The propagation of the damage induced by plasma to off-target cells could be explained by two mechanisms—the bystander effect and the abscopal effect. The bystander effect grants treated cells the ability to transmit signals to untreated neighbouring cells (in contact or not with the treated cells) to induce biological changes in them. These signals can be transmitted using soluble molecules, communication junctions (ion channels, pannexins, gap junctions and chemical synapses), occluding junctions (tight junctions) and anchoring junctions (adherens, desmosomes, focal adhesions, and hemidesmosomes) (Table 2). The expression of soluble cues, such as chemokines, cytokines, and growth factors, are directly affected by the oxidative stress induced by plasma, as demonstrated before in cancer and immune cells [134,194,195]. In particular, tumour cells exposed to plasma or plasma-treated medium can induce a bystander effect that leads to cell death in the untreated neighbouring population, a process mediated by the generation of secondary ^1^O_2_ and inactivation of membrane-bound catalase, as discussed in Section 2 [60,97]. Previous studies have reported that gap junctions can propagate cell death signals by passing Ca^2+^ ions from apoptotic to non-apoptotic neighbouring cancer cells [196], which could also explain the effect of plasma [197].

The abscopal effect grants treated cells the ability to evoke a response in cells that are at distant sites from the treated region, i.e., a systemic response that involves the immune system for its effect at sites distant from the treatment site [198]. This is the case of immunogenic cell death, a mechanism observed in plasma-treated cancer cells that induces the expression or release of danger-associated molecular patterns to activate a robust adaptive immune response against tumour cells [35,199,200,201]. In the same way, plasma has shown to suppress the growth of treated and non-treated remote melanoma tumours in mice, which could indicate the participation of the immune response upon plasma treatment [34]. The messages sent between cells can also be transmitted via tunnelling nanotubes and extracellular vesicles (EVs) that allow cargo exchange between cells located at short or long distances. Interestingly, plasma was able to hinder the formation of extracellular vesicles produced by ovarian cancer cells [195], which cargoes could stimulate pro-oncogenic responses and therapy resistance in neighbouring cancer cells. This is particularly important in the TME, as the crosstalk between malignant and stromal cells participates in the regulation of proliferation, angiogenesis, evasion of cells of the immune system and cell recruitment. For example, DNA present at the surface of EVs can modify the ability of EVs to interact with the ECM, whereas oncogenes transferred as single- or double-stranded DNA can increase the production of specific proteins (such as those involved in the response to oxidative stress) in the recipient cells [202].

Despite the growing evidence of the functional impact of EVs in cancer development, it is still unknown whether or not plasma can modify the cargo of EVs secreted by cancer or other cells in the TME to prevent tumour progression, metastasis, or angiogenesis. In the same way, the novel mechanism of intercellular communication, named tunnelling nanotubes (TnTs), allows cancer cells to interact with other cell types present in the TME. This interaction brings a unique opportunity to cancer cells: the acquisition of special characteristics that enable them to spread into distant sites [252]. These connective structures link the cancer cell to any other cell type for the transference of cytoplasmic signals, mitochondria, microRNA, and other cellular components, including death signals [227]. A novel model of ROS-dependent TnT formation mechanism has been proposed, which could explain the restoration of the redox homeostasis through the intercellular exchange of mitochondria, but where high ROS levels would lead to the disruption of TnTs to isolate the apoptotic population [253]. The participation of TnTs in the response to plasma treatment is yet to be studied. There is a growing body of literature supporting the potential of plasma to modify the communication mechanisms between cells. However, a significant number of these studies correspond to the evaluation of plasma on keratinocytes, epithelial cells, or fibroblasts in the context of wound healing (Table 2). More studies are needed to understand how plasma modifies the mechanisms of communication in the TME.

### 4.2. Cell-to-ECM Communication

The hypoxic environment is one of the main factors which control the ECM remodelling, deposition and degradation in TME. Hypoxia is achieved by proteins such as hypoxia-induced factors 1 and 2 (HIF1 and HIF2) [254]. This in turn leads to remodel the ECM by overexpressing fibrous proteins (e.g., collagens, fibronectins, and laminins) depending on the local level of hypoxia. Among the fibrous proteins, the increased collagen deposition serves as the identification of ECM alteration that occurs in the TME. Approximately 90% of the ECM is composed of collagen; hence it is one of the primary players in the physical and biochemical properties of the TME, modulating the tumour cell signalling, polarity, and migration.

HIFs actively regulate the expression of intracellular (P4HA and PLOD) as well as extracellular collagen-modifying enzymes (LOX), which induce hydroxylation of proline and lysine residues in collagens [255]. Current post-translational modifications increase the thermal stability of the collagen triple helix. The latter spontaneously forms collagen fibrils by covalently crosslinking on hydroxylysine and lysine residues by collagen peptidase, after secretion into the extracellular space from the endoplasmic reticulum. In the final stage, LOX catalyses collagen fibrils by again cross linking fibrils via the lysine aldehyde or hydroxylysine aldehyde and forms collagen fibre. The higher stability and stiffness of collagen fibre is required for the progression of metastasis in tumour cells. Previous investigation results showed that remodelling and deposition of existing collagen fibres serve as a hallmark of tumour transition to metastasis in cancer cells [256]. The application of HIF as well as LOX-targeting drugs or antibodies inhibited metastasis in cancer cells [89,257,258,259]. The use of plasma in would healing has demonstrated beneficial effects [260]. Plasma-generated ROS directly or indirectly induce oxidation of ECM components, affecting the healing process of the wound. It was observed that short plasma treatment increased collagen production in the wound area [261]. This is most likely due to adequate oxidation and deposition of collagen fibres, which is facilitated by plasma exposure. In contrast, in cancer treatment, reactive species might deactivate collagen modifying enzymes (e.g., P4HA, PLOD and LOX) or disturb the function of HIF. This in turn could lead to the inhibition of collagen deposition in the TME, suppressing the metastasis of cancer cells.

Fibronectin (FN) is also one of the essential and major components of the ECM and it is involved in regulation of cell differentiation, adhesion, growth, and migration. Specifically, soluble cellular FNs assemble into fibrillar matrix by transmembrane protein CD93 and certain domains of this matrix bind to integrin, fibrin, collagen, fibulin and syndecan, forming a complex network in the ECM [262]. III_10_ domain (Arg-Gly-Asp sequence) of FN matrix attaches to the cell through the α5β1 and αVβ3 integrins on the cell surface. Thus, FN mediates the interaction between ECM molecules and the cell. Particularly, evidence indicated that the upregulation of FN promotes tumour growth, metastasis and drug resistance in many cancer cell types [263]. Therefore, FN also serves as a biomarker oblivious of cancer cells. Moreover, FN actively protects cells from drugs and radiation therapy, suppressing apoptosis by initiating a number of intracellular pathways [264,265]. It was determined that FN expression is relatively higher in the metastasis site in comparison with the primary tumour [266]. The therapeutic agents against FN resulted in reduction of tumour size [267,268,269]. Hence, FN is one of the major players in the TME; however, the effect of plasma-generated ROS on the FN has not been studied yet. Nevertheless, it was determined that plasma did not affect the expression of integrins, e-cadherin and EGFR [184]. According to atomic scale simulations, oxidized proteins become more flexible and solvent accessible [270,271]. Consequently, considerable conformational changes take place in the protein structure, altering proper signalling as well as functioning at the cellular level [270,272,273]. Probably, RONS induce oxidation of FN, preventing the formation of fibrillar matrix. In addition, the binding between oxidized FN and other ECM components (i.e., type V collagen, laminin, entactin, perlecan and integrin receptors) might become less favourable, disrupting the communication pathways between the ECM and the different transmembrane adhesion proteins of tumour cells [274]. Eventually, ROS delivered by plasma to the TME might inhibit metastasis and growth of cancer cells. In order to establish clear molecular level mechanisms of ROS interaction with the ECM and its role in cancer treatment, this topic needs to be further studied in detail by experiments and computer simulations.

## 5. Novel 3D in vitro Models to Explore the Effect of Plasma on the TME

The majority of studies on plasma treatments for cancer are carried out on cells propagated in two dimensions (2D) on flat surfaces. The findings obtained using conventional 2D cell culture models have provided vast insight on how plasma affects cancer cells in vitro. However, it has been demonstrated that cells cultured in 2D are often not representative of the cells present in tumours as they lack the cell-to-cell and cell-to-ECM interactions characteristic of the tumour microenvironment [275]. 3D culture models offer the opportunity to more closely resemble the complex architecture and interactions between cells and the tumour native environment [276]. Cells growing in 3D cultures have gene and protein expression profiles that simulate those of tumours in situ and affect cell morphology, metabolism, signal transduction, aggregation, response to stimuli and differentiation [276,277]. These unique features make 3D cultures a valuable tool to investigate the mechanisms of action of plasma in tissue-like constructs in vitro and bridge the gap between in vitro and in vivo.

There are several methods to build 3D cell cultures which provide different levels of complexity and insight into the response to treatment (Table 3). The current limitation of most of the in vitro culture methods used in plasma research is the excessive amount of liquid present during the treatment, which does not resemble the real conditions found when treating patients. This is particularly important for plasma sources with an active flow of gas toward the target sample, as in the presence of little or no liquid, the active gas flow could induce cell stress by dehydration in cell cultures [278].

**Figure 4:**
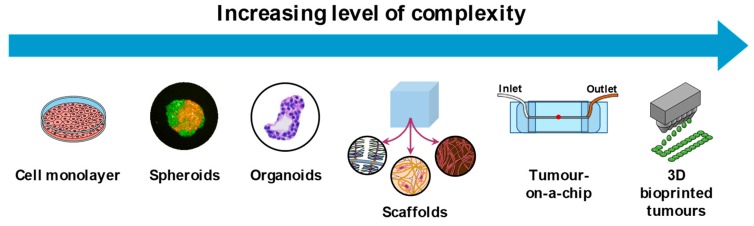
3D culture models for in vitro research. The arrow indicates the increasing level of complexity of each model. To date, in vitro plasma research has been performed using conventional 2D cultures, spheroids, and scaffolds, but other 3D models could be incorporated. Scaffolds formed by various ECM components and cells.

The main challenge for the plasma community is to adopt appropriate and relevant models that satisfy the requirements of the plasma source used (with little or no interference from excessive amounts of liquid) and the mode of treatment delivery (direct plasma application or use of plasma-treated solutions). Despite these limitations, the 3D culture models presented here can provide valuable insights in the response of cancer cells and the TME to plasma that are more translatable to conditions in patients than from conventional 2D cultures (Figure 4). In addition, these models contribute to reducing the number of animals used in scientific research, while still providing insightful data for the development of cancer therapies.

### 5.1. Spheroids

One of the most suitable models for the study of anticancer treatments are the 3D spheroids, as they can reproduce key features of solid tumours in vivo, such as physical communication and signalling pathways, ECM deposition, gene expression, and response to anticancer therapies. Spheroids can be formed in a few days using cancer cells exclusively (homotypic) or in combination with endothelial cells, fibroblasts, or immune cells (heterotypic). By modulating the ratio of cancer to stromal cells, it is possible to mimic the cellular heterogeneity of solid tumours. Spheroids have a highly proliferative external layer, as these cells have access to nutrients and oxygen, whereas the middle and inner core consists of senescent or necrotic cells due to the hypoxic environment within the spheroid. The lack of nutrients in the hypoxic core promotes the conversion of pyruvate to lactate (Warburg effect) to obtain energy, which decreases the pH of the tissue, as observed in solid tumours [279]. 3D spheroids treated with plasma (directly and indirectly using plasma-treated liquids) presented cell death, DNA damage, cell cycle arrest, and hindered proliferation and cell migration [28,35,280,281,282,283]. While cell death was observed in the outer layer of the spheroid, cells in the spheroid core were in a state similar to cell arrest, suggesting that the effect of plasma can penetrate into the tissue to affect non-superficial cells [28]. This could be related not to the direct effect of plasma-derived ROS into the tissue, but to the propagation of oxidative stress signals and oxidation products to neighbouring cells, as discussed in Section 4.1. In combination with 2D and in ovo approaches, this model has been used to demonstrate that plasma treatment does not evoke a metastatic behaviour in pancreatic cancer cells, an encouraging finding that supports the application of plasma in oncology [284]. Furthermore, it has been demonstrated that the combination of plasma with the antineoplastic drugs doxorubicin, epirubicin, and oxaliplatin enhanced their cytotoxic effect in melanoma cells in spheroids, possibly due to the upregulation of the organic cation transporter SLC22A16 upon plasma treatment [285]. These findings are particularly relevant, as they suggest that plasma has the potential to improve the delivery and cytotoxic effect of current antineoplastic drugs. Studies using co-culture spheroids could provide further insight in how plasma affects the stromal cells present in the tumour and their role modulating the response of cancer cells to plasma.

### 5.2. Organoids

These 3D constructs can be developed from adult stem cell-containing tissues (isolated organ progenitors), single adult stem cells, embryonic stem cells, or induced pluripotent stem cells from normal or malignant tissue. In this construct, cells can differentiate into multiple, organ-specific cell types to form structures similar to that of organs in vivo and functions specifically to the parent organ [286]. Organoids are particularly relevant for the study of toxicity and efficacy of anticancer treatments, as they can effectively recapitulate the treatment response of in vivo cancers with high sensitivity and specificity for chemotherapeutics [286]. The model can be further improved by the addition of stromal or immune cells to the culture to generate a more complete organotypic culture system. To date, organoids have not been used for plasma research, probably due to the high costs and time-consuming protocols. However, this tool could help developing effective plasma treatments for cancer and facilitate the transition into the clinic.

### 5.3. Scaffolds

3D scaffolds can be made of organic (collagen, gelatin, fibrinogen, hyaluronan, alginate, silk, etc.) or synthetic polymers (polyethylene glycol, poly-D,L-lactic-co-glycolic acid, and polyglycolic acid) that provide structural support to cell adhesion, proliferation and tissue development. In scaffold-based 3D cultures, the cell behaviour is influenced by the chemical and physical properties of the material used, such as porosity, stiffness, and stability in culture [275]. Scaffolds can be packed with growth factors and short-peptide sequences derived from ECM components that can improve cell adhesion and proliferation [287], as well as to serve of ROS reservoir for the passive delivery of oxidative stress to target cells [288]. In addition, natural tissue scaffolds called “decellularized ECMs” (dECM) can be prepared from native or regenerated tissues in vitro by removing cells with enzymes, detergents, or hypertonic solutions. Tissue-derived dECM has similar composition, bioactive signals, and mechanical properties of the native microenvironment, whereas cultured cell-derived dECM can be prepared in large scales and its composition can be modulated by the culture conditions [289]. Scaffolds are useful substrates to study the effect of plasma-derived ROS on growth and invasion of cancer cells. Previous studies have shown that plasma can change the biophysical properties of polymers, as it can enhance the polymerization and biophysical stimulation of biomaterials used for bone and cartilage regeneration [290,291]. Thus, it could be expected that plasma would oxidize or modify the properties of these scaffolds in 3D cultures, therefore providing relevant information about the effect of plasma on the ECM and cells of the TME under oxidative stress.

### 5.4. Microfluidics-Based Tumour Models—Tumour-on-a-Chip

This microfluidics model—not yet used on biomedical plasma research—is suitable for the study of plasma-treated solutions (alone or in combination with other compounds) and not for direct plasma applications. Tumour-on-a-chip is a microfluidic cell culture prepared in porous plastic, glass, or flexible polymers that recapitulate in vitro the structure, function, and mechanical properties of organs in vivo, by modifying cellular, molecular, chemical, and biophysical factors in a controlled fashion [292]. This model allows manipulation of fluid temperature, flow pressure, shear stress, and oxygen and nutrients gradients required to mimic the processes occurring in vivo. This system can include tumour, stromal and endothelial cells, which allows the formation of vasculature and the study of angiogenesis, lymphoangiogenesis, intravasation, extravasation, and metastasis [293]. This controlled model allows experimenting with various combinations of molecular, biophysical, and chemical parameters to study tumour progression, invasion, migration, and epithelial-mesenchymal transition in response to treatment. This model is more difficult to use due to the low throughput, time needed to run an assay and high level of complexity to perfectly tune all the parameters needed to achieve an optimal model, in addition to the associated costs [292]. However, the potential of microfluidics-based models in plasma research is broad, as it could be used in multiple cell types to determine the therapeutic effect and toxicity of plasma-treated solutions before going into clinical trials.

### 5.5. 3D Bioprinted Tumour Model

This technique allows the formation of complex tissues with a variety of cell types organized in a defined spatial architecture in a scaffold-free environment [294]. 3D bioprinted tumours in vitro can be used to test a variety of responses in tissues exposed to treatment using cell lines and patient-derived tumour cells. One of the main benefits of this model is the possibility to generate large, heterotypic tumour tissues with cancer and stromal cells with a specific spatial orientation that interact in a complex and defined microenvironment. This model can recapitulate the TME heterogeneity and vasculature of in vivo tumours and provide valuable information on the crosstalk between malignant and stromal cells in response to specific treatments (intrinsic and extrinsic signals) [295]. The sophisticated technique has enabled many laboratories to develop biologically functioning 3D in vitro models of liver, kidney, skin and malignant tumours, and is used as a drug screening tool [296]. 3D bioprinted tumours could be advantageous for the study of in vitro solid tumours in response to plasma therapies in the future, but to date there are no reports on its use in the field of biomedical plasmas.

## 6. Perspectives and Conclusions

The complex mix of ROS delivered by plasma is able to induce multiple modifications both in the cancer cells as well as in the cells and molecules present in their vicinity. Considering the possible application of plasma for therapeutic purposes in cancer, it is necessary to understand the interaction between plasma-derived ROS, the malignant cells and the TME. Specifically, it is important to understand how cells communicate the signals evoked by plasma-derived ROS and how could plasma affect these mechanisms. As discussed in this review, there are multiple cellular and acellular components that directly affect the response to treatments and therefore they should be considered in the experimental approaches used to investigate the effect of plasmas in cancer. However, as the field of biomedical plasmas is still developing, there are still many unknowns that need to be addressed. In the past few years, there has been an increase in the number of publications using more complex 3D cell culture models, alone or in combination with other cells of the TME. The advantage of adopting such technologies is the possibility to mimic the response to treatment obtained in real solid tumours, such as the effect of plasma on cells of the immune system, stromal cells, ECM components, secretion of soluble factors, and alteration of mechanisms of cell communication, among others. Furthermore, it is possible to use these technologies to assess the toxicity of plasma in normal cells and confirm the selective nature of plasma therapy, as this is paramount for the application of plasma in patients. To date, there is a limited number of clinical trials done in cancer patients as palliative (head and neck cancer) and curative treatments (melanoma and ovarian cancer) [19]. To move forward in this field, it is necessary to develop standardized protocols and safety guidelines for plasma that acknowledge the role of the TME in the outcome and reduce the risk of secondary effects in healthy cells. Another key point is the delivery of the treatment to hard-to-reach regions inside the body, or the need of multiple applications of plasma in regions accessible only during surgical procedures. The development of small, flexible plasma probes that can be used in less invasive procedures like endoscopy or laparoscopy (e.g., flexible argon probes similar to those used for plasma coagulation and electrosurgery), or the use of plasma-treated solutions, could facilitate the delivery of plasma and the translation of this technology into the clinic. In this spirit, considering the implementation of adequate experimental approaches and the increasing collaborative work done between plasma scientists and immunologists, oncologists, and engineers, we foresee an expansion of the current knowledge on biomedical plasmas for cancer in the near future.

The work done using the conventional 2D cultures and the more relevant 3D in vitro models can be significantly strengthened by in silico modelling approaches. The paradigm of the underlying mechanisms of selective anticancer plasma treatment presented in [58] is based on apoptosis-induction as a consequence of a manipulation of the communication between the cancer cells and the ECM, resulting in apoptosis induction, and a subsequent cell-to-cell communication, where the apoptosis signal is transferred to adjacent cells. More knowledge about these mechanisms can be achieved by experimental studies, but a parallel avenue is a theoretical approach where the spatial and temporal dynamics of the key species involved are analysed by mathematical modelling. The theoretical approach is so far novel and will require significant efforts to fully capture the complexity of the proposed signalling pathways, but has successfully been used to increase the knowledge of similar mechanisms, such as those of the cell antioxidant defence, as well as other sorts of cell signalling mechanisms [297,298,299,300,301,302,303,304,305,306,307,308,309]. A key advantage of mathematical modelling is the possibility to probe a system within regimes that are not feasible experimentally. However, there is a lack of information on the molar concentrations of the involved enzymes and ROS in the ECM (i.e., not the total concentration of the cell), proper description of the enzyme mechanisms and limited access to the kinetic parameter values. Without this essential information, it is challenging to develop a predictive mathematical model of the spatial and temporal dynamics of the involved species. For most enzyme reactions, information about mechanisms and kinetic parameter values are from experiments, which do not resemble the true in vivo situation. Furthermore, there are most likely a number of regulation mechanisms, such as enzyme inhibition by the reaction products (or other species), for which there exist no experimental data. The pH-dependency of enzyme activities is yet another important factor that is difficult to take into account in a mathematical model. Lastly, there is little information on the difference between the catalytic action of membrane-bound enzymes and enzymes that are free in solution. From Pólya’s theorem on random walks—stating that a random walker confined in one or two dimensions is guaranteed to find a stationary target, while a random walker in three dimensions might not—it could be argued that membrane-bound enzymes would have an increased catalytic action compared to enzymes that are free in a solution (given that the substrate exhibits an affinity for the cell membrane). However, Pólya’s theorem only concerns the probability to find a target and not the actual diffusion time; in reality the hypothetical catalytic advantage of a membrane-bound enzyme crucially depends on the ratio of surface to bulk-phase diffusion coefficients. Furthermore, as is the case with covalently bound enzymes—like catalase in the extracellular compartment of cancer cells [90,91,95,99,101,102,310,311]—the covalent attachment itself may also modify the enzyme. Despite the existing limitations, the mathematical modelling of cell signalling pathways in the ECM of cancer cells is a fruitful approach and an excellent complement to experimental studies, to increase the understanding of the underlying mechanisms of selective anticancer effects of plasma.

Understanding how plasma modulates the mechanisms of communication between cancer cells and the TME and the concomitant modifications caused to the TME is of outmost relevance to develop plasma therapies for cancer that can be translated into the clinic. 

## Figures and Tables

**Table 1:** Overview of the main components of cell-to-ECM communication mechanisms and their role in the response to plasma treatment.

Molecule	Physiological Role(s)	Reported Response to Plasma	Redox Changes and Functional Consequences
**ECM components**			
Hyaluronan	Regulates cell behaviour interacting with CD44 [167]. Auto- and paracrine signalling between CAF and tumour cells [163]	Unknown	ROS can depolymerize and fragment HA aggregates [165]
Collagen (COL)	Major ECM component, supports cell movement through ECM [168]	Oxidized amino acids, broke H-bonds [153], and loosen COL I structure [154]. Fibroblast activation to produce COL [169]	ROS induce overproduction, deposition and remodelling of COL to generate stiffer tissue ECM in tumours [104,170]
Laminin (LM)	Glycoprotein, important in cell differentiation, migration, and adhesion [171]. Cell–cell and cell-ECM interaction	Increased expression in wound bed region in mice [172]. Enhanced adhesion, growth, and viability in plasma-treated LM-modified PCL* [173]	ONOOH and HOCl induce nitration, oxidation, and chlorination of LM residues [174,175,176]. Modification of self-polymerization and cell adhesion sites, which modulates the ECM structure
Fibronectin (FN)	Involved in cell adhesion, growth, migration, and differentiation	Increased FN expression in THP-1 cells by kINPen [131]. Induced FN formation in activated fibroblasts [169]	ROS oxidize FN [166]. HOCl causes FN oxidation, which modulates cell adhesion, proliferation, and mRNA expression [177,178]
Elastin	Entropic elastic behaviour, its stretch is limited by its association with collagen [179]	Fibroblast activation in mouse skin to produce elastin [169]	ROS destroy elastin network integrity, influence production of ECM proteins [180]. ONOOH induces tyrosine nitration and crosslinking in topoelastin, alters its structure, function, and changes matrix assembly [181]
**Adhesion to ECM**			
Focal adhesions (FA)	Complex protein assemblies, bind cells with ECM via actin/integrin links. Mediates mechanical and biochemical “outside-in” and “inside-out” signalling [182]. Cell-ECM and cell–cell adhesion	Increased FA size in WTDF3 mouse fibroblasts [183], expression of α_2_-integrin/CD49b, β_1_-integrin/CD29 [184], β_1_-integrin [185] and FA proteins in HaCaT cells [42]. Activated β_1_-integrin in WTDF3 mouse fibroblasts [183]. Reduced α5- and β_1_-integrin in fibroblasts, PAM cells [186,187]	Oxidative stress activates FA kinase, accelerates cell migration [188]. Integrin-linked kinase (ILK) signalling via PKB/Akt can suppress apoptosis and anoikis [189]. ILK required to maintain redox balance [190]. NRF2 mediated oxidative stress response [191]
Hemidesmosomes (HD)	Integrin-based adhesive junction [192]. Cell-ECM and cell–cell adhesion	Upregulation of proteins related to HD assembly by plasma [42]	HD disruption facilitates cell detachment and migration [193].
**Catalytic enzymes**			
Catalase	Membrane-associated catalase protects cancer cell from apoptosis-inducing signalling pathways	Inactivation through reaction of ^1^O_2_ with histidine in the active site [59]	Generation of •OH leads to lipid peroxidation and induction of apoptosis. Generation of ^1^O_2_ inactivates catalase at adjacent cells [59]
Superoxide dismutase (SOD)	Membrane-associated SOD protects catalase from inactivation by O_2_•^‒^	Inactivation through reaction of ^1^O_2_ with histidine in the active site [59]	Increased concentration of O_2_•^‒^ and subsequent enhanced generation of •OH [59]
Peroxidase (POD)	Pathogenic resistance	Unknown	N/A
NADPH oxidase 1 (NOX1)	Cell proliferation	Unknown	N/A

* PCL = polycaprolactone.

**Table 2:** Overview of the main inter- and intracellular mechanisms of communication and their role in the response to plasma treatment.

Molecule	Physiological Role(s)	Reported Response to Plasma	Redox Changes and Functional Consequences
**Communication junctions**			
Ion channels	Ca^2+^-permeable and voltage-independent cation channels. Include transient receptor potential (TRP) channels. Auto- and paracrine cell–cell communication	Activated intracellular Ca^2+^ influx through TRP channels [203]. Induced Ca^2+^ release by ER* and mitochondria needed to induce senescence in melanoma cells [204]	ROS affect channel function, structure and downstream signalling pathways [205]. Can sense lipid oxidation [206]. Increased intracellular [Ca^2+^] by TRPC3 and TRPC4 leads to cell death upon oxidative stress [207]. H_2_O_2_ oxidizes TRPM2 and induces chemokine production [208]. TRP7 blockade induces apoptosis [209]
Pannexins (Panx)	Transmembrane proteins, form channels for the release of ATP and other metabolites [210]. Auto- and paracrine cell–cell communication	Unknown	Oxidative stress regulates Panx channel activation; ATP, ADP, and AMP release for apoptotic cell clearance [210]. Overexpression of Panx1 in cancer [211], its inhibition reduces tumour growth and invasiveness [212]. NO may inhibit Panx1 current [213]
Extracellular vesicles (EVs)	Secreted exosomes, microvesicles and apoptotic bodies [214]. Interact with adjacent or distant cells [215]. Para-, auto-, exo- and endocrine cell–cell communication	Increased number of EVs released by THP-1 and PMN* [216]. Less EVs produced by plasma-treated OVCAR-3 and SKOV-3 ovarian cancer cells [195]. Induced formation of apoptotic bodies [26,217,218]	Tumour cells produce high number of EVs with altered redox balance and ROS levels. EVs can scavenge/produce ROS and modify ROS content in target cells [219]. EVs involved in tumour development and metastasis [214]
Gap junctions (GJs)	Connect cells for electrical and metabolic (sugars, ions, amino acids, nucleotides) communication [220]. Auto- and paracrine communication	Plasma-generated ROS and intracellular ROS produced upon plasma treatment triggered bystander effect and damaged GJs [197]	Bystander effect: GJs can transmit ROS and cell death signals to neighbouring cells [196,221]
Connexins	Form gap junctions, transfer ions, small messengers, and metabolites. Forms hemichannels that communicate intra- and extracellular spaces [222]	Destroyed structure of connexins’ N-terminal tail [197]. Temporary loss of cell–cell contact [223]. Reduced Cx43 connexin expression in epithelial cells, transient increase of Cx43 in fibroblasts [187]	Redox status modulates the opening/closing and permeability of connexin hemichannels to NO and large molecules [224]
Tunnelling nanotubes (TnTs)	Long, filamentous, actin-based structures, connect cells to transfer drugs, organelles, nucleic acids, and proteins [225]. Cell–cell communication	Unknown	High H_2_O_2_ levels induce TnTs formation [226]. Propagation of death signal Fas ligand through TnTs between T cells [227]. TnTs mediate mitochondria transfer to rescue cells on oxidative stress [228]. Increased number of TnTs upon high oxidative stress [229]
Tight junctions (TJs)	Restrict diffusion based on size and charge to maintain homeostasis. TJs maintain cell surface polarity [230]. Cell–cell communication	Disrupted tight junctions in epithelial cells and caused retraction of Zonula occludens ZO-1 protein from cell membrane [231]	High doses of NO and H_2_O_2_ increases paracellular permeability in epithelial cells [232]
Claudins	Main structural TJs proteins. Block lipid and protein diffusion, ease transference of small ions [233]	Downregulated expression by repetitive exposure to plasma-treated medium [234]	ONOO^‒^ could interfere with claudin function [235]. Lipid peroxidation [236] and H_2_O_2_ can disrupt tight junctions [237]
Occludins	Contribute to TJ stability and barrier function [238]	Downregulated expression by repetitive exposure to plasma-treated medium [234]	Oxidative stress reduces occludin oligomerization [239], interaction with other TJ proteins and barrier tightness [240]. H_2_O_2_ induces occludins cleavage [241]; NO abolishes its immunoreactivity and redirects it to cytoplasm [240]
**Anchoring junctions**			
Adherens	Homophilic lateral cell-to-cell adhesion via cadherin/catenin complex, transmit mechanical forces between cells, regulate signalling and transcription [242]. Required for TJs assembly [233]	Decreased E-cad expression [185,187]; function modulation, internalization in HaCaT cells [243]. Decreased E-cad in mice epidermis cells [243]. Increased E-cad expression in wounds of rats [244] and β-catenin expression in keratinocytes [234]	ROS selectively disrupts cadherin/catenin complexes [245,246], modulate receptors involved in cell-matrix and cell–cell adhesion [247]. Loss of E-cadherin activates EMT [248].
Desmosomes	Intercellular junctions, link cells and stabilize the tissue structure [249]. Cell–cell adhesion	Increased the number of desmosomes in wounds [105]	ROS induce PKP3 phosphorylation, pPKP3 release from desmosome and desmosome instability [250]. Desmosomes are intracellular signal transducers in Wnt pathway [251]

* ER = endoplasmic reticulum; PMN = polymorphonuclear leukocytes.

**Table 3:** Advantages and disadvantages of in vitro 3D culture models for plasma research.

3D Culture Models	Main Feature	Advantages	Disadvantages	Suitable Plasma Treatment
**Spheroids**	Self assembly	Formed from cell lines	Simple architecture	Direct and indirect
		Recreates gradients of nutrients/oxygen	Not all cell lines form spheroids	
		Easy to generate	Static conditions	
		Uniform size Reproducible		
		High-throughput	Plasma treatment in presence of liquid	
		Allows co-cultures		
**Organoids**	Capable of self-renewal and self-organization	Formed from primary cells	Require validation to identify outgrow of unwanted cells (normal/cancer cells)	Direct and indirect
		Requires small amount of tissue	Requires access to human samples	
		Resembles complexity, architecture, gene expression from in vivo tumours	More expensive	
		Can be transplanted into mice	Static conditions	
		Allows co-cultures	Plasma treatment in presence of liquid	
**Scaffolds**	Naturally-derived ECM components or synthetic polymers	Formed from primary cells or cell lines	Batch-to-batch variability of natural matrixes	Direct and indirect
		Resemble mechanical forces in tumours		
		Versatile	Synthetic matrixes can be expensive	
		Diffusion gradients		
		Very reproducible	Might require complex cell retrieval/imaging methods	
		Allow co-cultures		
**Tumour-on-a-chip**	Spatiotemporal control of chemical/physical properties	Formed from primary cells or cell lines	More expensive	Indirect only
		Resembles diffusion gradients/perfusion	Requires special equipment	
		Highly sensitive		
		Vascularized	Difficult to scale up	
		Allow co-cultures		
**3D-bioprinted tumours**	Precise control of biomaterials, cells, and biological factors	Formed from primary cells or cell lines	More expensive	Direct and indirect
		Recreates natural function and structure	Requires special equipment	
		High-throughput		
		Vascularized	Needs optimization	
		Allow co-cultures		
